# The mitochondrial genome of *Asyndetus clavipes* (Diptera: Dolichopodidae)

**DOI:** 10.1080/23802359.2021.1911714

**Published:** 2021-04-15

**Authors:** Juan Wang, Yuyan Li, Lisheng Zhang, Mengqing Wang

**Affiliations:** a College of Plant Protection, Shanxi Agricultural University, Taigu, Shanxi, China; b Institute of Plant Protection, Chinese Academy of Agricultural Sciences, Beijing, China

**Keywords:** Mitochondrial genome, Dolichopodidae, Diaphorinae, phylogenetics

## Abstract

The long-legged fly *Asyndetus clavipes* belongs to the subfamily Diaphorinae of Dolichopodidae. The mitogenome of *A. clavipes* was sequenced, the first representative complete mitogenome from this subfamily. This mitogenome is 15,385 bp in size, includes 13 protein-coding genes, 22 transfer RNAs, and two ribosomal RNAs. All genes have the same location and coding strand as in other published species of Dolichopodidae. Nucleotide composition is biased toward A and T, which together made up 78.6% of the entire genome. Bayesian inference strongly supported the monophyly of Empidoidea, Empididae and Dolichopodidae, with the phylogenetic relationships within Empidoidea: ((Dolichopodinae + Neurigoninae) + Diaphorinae) + ((Trichopezinae + (Empidinae + Oreogetoninae)) + Ocydromiinae).

## Introduction

*Asyndetus* Loew, 1869 belongs to the subfamily Diaphorinae. It is a large genus of Dolichopodidae, worldwide distributed with 102 species (Yang et al. 2006, 2011; Wang et al. 2007).

The adult specimens of *A. clavipes* used for this study were collected in Meitan County of Guizhou Province in China in 2017 by Mengqing Wang and identified by Mengqing Wang. Specimens were deposited in the Natural Enemy Insects Museum (Accession Number: NI2017-10) of the Institute of Plant Protection, Chinese Academy of Agricultural Sciences (IPPCAAS) (Room 307, Plant Protection Building). Total genomic DNA was extracted from a whole-body (except the head) specimen using the QIAamp DNA Blood Mini Kit (Qiagen, Germany) and stored at −20 °C until needed. The mitogenome was sequenced in BeiRuiHeKang biotechnology company used NGS. 1 μg of genomic DNA was used to generate libraries with an average insert size of 350 bp, which were sequenced using the Illumina NovaSeq 6000 platform (Berry Genomics, Beijing, China) with 150 bp paired-end reads on one sample per flow-cell lane. A total of 24,471,684 raw paired reads were generated. The quality of all sequences was checked using FastQC (http://www.bioinformatics.babraham.ac.uk/projects/fastqc). Clean reads were assembled and annotated using the MitoZ v2.4 pipeline (Meng et al. 2019).

The complete mitogenome of *A. clavipes* is 15,385 bp (GenBank accession number: MT949690) and encodes 13 PCGs, 22 tRNA genes, and 2 rRNA genes. All genes have the same location and coding strand as in other published species of Dolichopodidae (Qilemoge, Gao, et al. 2018; Qilemoge, Zhang, et al. 2018; Hou et al. 2019; Qilemoge et al. 2019, 2020). Nucleotide composition is biased toward A and T, with 78.6% A + T content (A = 40.5%, T = 38.1%, C = 12.5%, G = 8.9%). The A + T content of PCGs, tRNAs, and rRNAs was 77.3, 77.6, and 81.8%, respectively. Seven PCGs (*NAD2*, *COI*, *ATP8*, *NAD3*, *NAD5*, *NAD4L*, and *NAD6*) initiate with ATT codons, and six PCGs (*COII*, *COIII*, *ATP6*, *NAD1*, *NAD4*, and *CYTB*) initiate with ATG codons. All PCGs (12 of 13) use the typical termination codons TAA while *NAD4* uses TAG.

**Figure 1.**
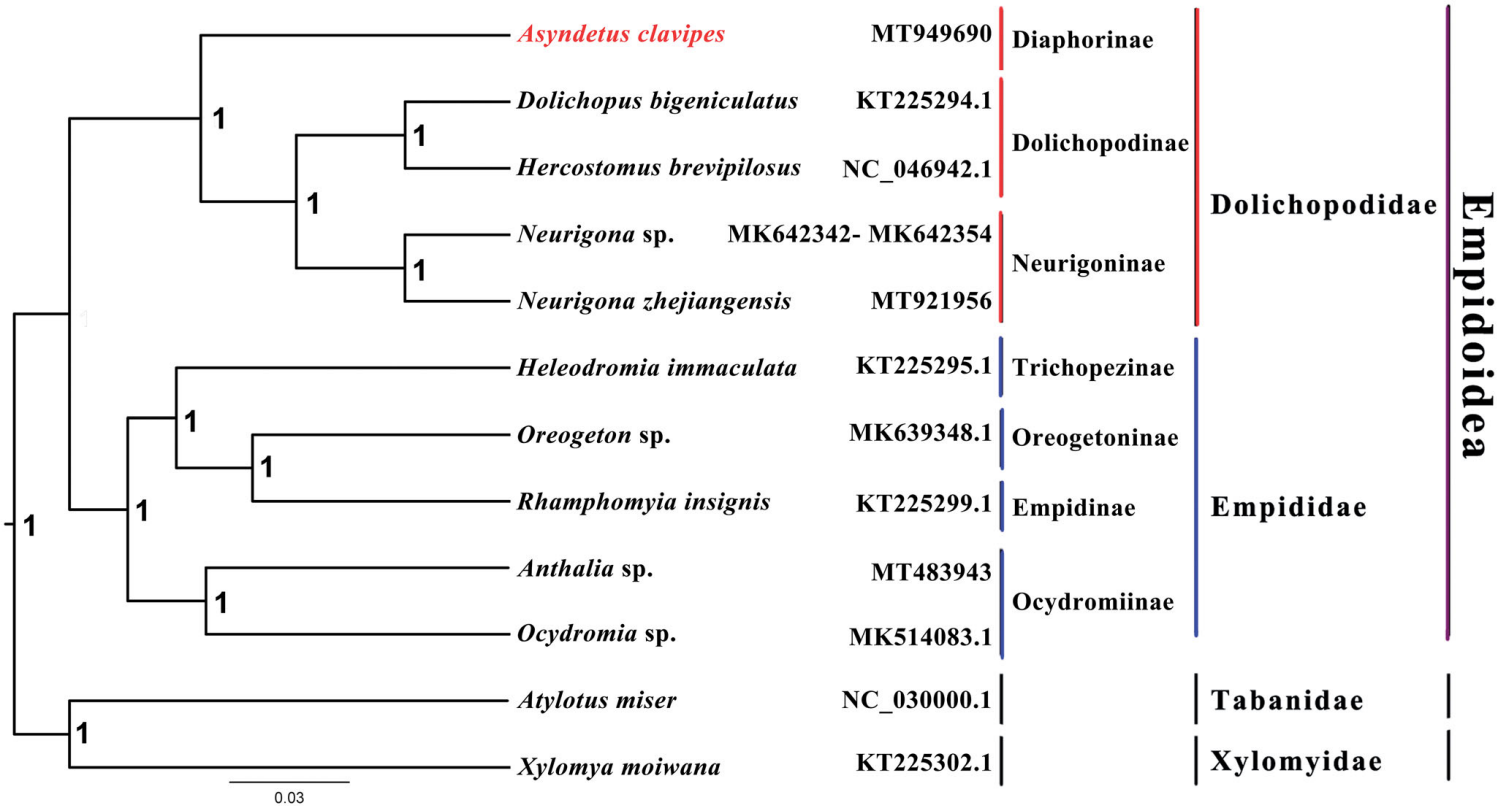
Bayesian phylogenetic tree of 12 Diptera species. The posterior probabilities are labeled at each node. Genbank accession numbers of all sequences used in the phylogenetic tree have been included in figure and corresponding to the names of all species.

Phylogenetic analysis was performed based on the nucleotide sequences of 13 PCGs from 12 Diptera species. Sequences were aligned using MAFFT v7.313 (Katoh and Standley 2013), and the Bayesian Inference (BI) tree was constructed with MrBayes 3.2.6 (Ronquist et al. 2012), which was run for 2,000,000 generations and sampled from every 100 generations. The CAT + GTR model selected by ModelFinder (Kalyaanamoorthy et al. 2017) was applied. Bayesian posterior probabilities were calculated after discarding the first 25% of the trees. The phylogenetic result strongly supported the monophyly of Empidoidea, Dolichopodidae, and Empididae. Monophyletic Dolichopodinae, Neurigoninae, and Diaphorinae were grouped as a monophyletic Dolichopodidae, which was a sister group to a monophyletic Empididae that consists of Oreogetoninae, Empidinae, Trichopezinae, and Ocydromiinae in this study (Figure 1). The monophyly of Empididae and Dolichopodidae is consistent with previous phylogenetic results (Wang et al. 2016). The mitogenomic data of *A. clavipes* could provide important information for further studies of Dolichopodidae phylogeny. Further studies are needed to sequence more species from the subfamily Diaphorinae, as well as other subfamilies, which will enhance our understanding of molecular phylogeny in Dolichopodidae.

## Data Availability

The genome sequence data that support the findings of this study are openly available in GenBank of NCBI at (https://www.ncbi.nlm.nih.gov/) under the accession no. MT949690. The associated BioProject, SRA, and Bio-Sample numbers are PRJNA681494, SRR13170302, and SAMN16954843, respectively.
